# Discontinuation of the development of remimazolam for ICU sedation in Japan: background and rationale

**DOI:** 10.1186/s40560-026-00849-8

**Published:** 2026-01-21

**Authors:** Junichi Saito, Kazuyoshi Hirota

**Affiliations:** 1 Department of Anesthesiology, Hirosaki University Graduate School of Medicine, 5 Zaifu-Cho, Hirosaki, 036-8562 Japan; 2 Department of Anesthesiology, Aomori Prefectural Central Hospital, 2-1-1 Higashi-Tsukurimichi, Aomori, 030-8553 Japan; 3 Department of Perioperative Stress Management, Hirosaki University Graduate School of Medicine, 5 Zaifu-Cho, Hirosaki, 036-8562 Japan

**Keywords:** Remimazolam, Carboxylesterase-1, An ultra-short-acting benzodiazepine, Pharmacokinetics, Intensive care units

## Abstract

Remimazolam, an ultra-short-acting benzodiazepine rapidly metabolized by carboxylesterase-1, was developed as a promising alternative for ICU sedation. It was anticipated to overcome the unpredictable accumulation associated with midazolam and the hemodynamic/metabolic risks of propofol, offering superior hemodynamic stability and potential benefits in reducing postoperative delirium. Its ultra-short, predictable half-life positioned it as an ideal candidate for facile titration in critically ill patients. However, the trajectory of remimazolam's development for long-term ICU sedation faced a critical setback in Japan. Based on results from the ONO-2745-04 Phase II trial conducted on mechanically ventilated postoperative patients, the development program for the ICU indication was halted in 2013. The central safety concern was the unexpected pharmacokinetic failure observed in a subset of patients receiving continuous infusion for 24 h or longer. Specifically, this subgroup exhibited plasma concentrations of the parent drug far exceeding predicted levels, resulting in significantly delayed awakening and recover. This observation directly challenged the fundamental non-accumulating advantage of the drug. The mechanism is hypothesized to be compromised carboxylesterase-1 activity due to severe critical illness, systemic inflammation, or organ dysfunction—conditions that impair the very non-organ-dependent clearance pathway the drug relies upon. While international experience continues to validate the safe and effective use of remimazolam for short-to-medium-term ICU sedation, the Japanese experience serves as a critical clinical warning. It underscores that even drugs with inherently favorable pharmacokinetic profiles are susceptible to unpredictable parent drug accumulation in the highly heterogeneous and physiologically compromised ICU population during prolonged infusion. Therefore, extreme caution and individualized dosing strategies are warranted for remimazolam use in critically ill patients, especially those with severe systemic dysfunction.

## Background

The management of pain, agitation, and delirium remains a cornerstone of intensive care unit (ICU) practice [1]. Although midazolam and propofol have long served as workhorse sedatives, their limitations (particularly the unpredictable clearance and accumulation of midazolam in critical illness, and the risk of propofol infusion syndrome) have driven the search for alternatives. Remimazolam, a novel ultra-short-acting benzodiazepine, was developed to address these issues. Its rapid and predictable onset and offset profile, attributed mainly to metabolism by an enzyme encoded by the CES1 gene, i.e., carboxylesterase-1 (CES-1), positions it as a potentially ideal agent for the rapid control and titration that are required in ICU settings [2]. Despite this promising pharmacological profile, remimazolam's journey towards widespread ICU adoption has been complex, notably involving a halt in its clinical development for ICU indications in certain regions, including Japan.

This review, targeted at intensive care specialists, summarizes the current evidence surrounding remimazolam, focusing on the reasons behind the Japanese development strategy and a critical examination of remimazolam's pharmacokinetic (PK) profile, specifically addressing the persistent question of drug accumulation in critically ill patients.

## The clinical trial landscape in Japan: understanding the discontinuation of remimazolam’s development for ICUs

The development trajectory of remimazolam has been inconsistent across different geographies. Although several studies, predominantly from Asian and European centers, have explored the use of remimazolam in ICU settings [2–7], the domestic Japanese development program for the applications of remimazolam for ICU sedation was discontinued in 2013. This decision was based mainly on the outcome of the Japanese Phase II clinical trial, ONO-2745–04, which investigated continuous remimazolam infusion as a sedative for postoperative patients requiring mechanical ventilation.

The ONO-2745–04 trial targeted patients undergoing elective surgery who were expected to require mechanical ventilation postoperatively for ≥ 6 h. After the patients' transition to the ICU and the discontinuation of all prior sedatives, continuous remimazolam infusion was initiated at 0.1, 0.25, or 0.5 mg/kg/h when the patient's Richmond Agitation-Sedation Scale (RASS) score was between −1 and −2. The dosage was subsequently titrated to achieve the optimal sedation depth, defined as RASS − 1 to − 4, with a maximum permitted infusion rate of 2 mg/kg/h. The treatment duration was limited to 7 days or until the cessation of mechanical ventilation, whichever came first. The interim analysis of the trial's results demonstrated that the primary endpoint of achieving the optimal sedation depth was highly successful: the target sedation depth (RASS − 1 to − 4) was maintained for ≥ 70% of the total drug administration time in 97% (35 of 36) of the patients. This confirmed remimazolam’s efficacy in providing the necessary level of sedation.

Despite these strong efficacy data, the trial was halted due to significant safety and pharmacokinetic (PK) concerns, particularly with prolonged administration. Among the patients who received a continuous infusion for > 24 h, a subset exhibited plasma concentrations far exceeding predicted levels. More critically, all of the patients in this > 24-h infusion group required ≥ 1 h complete awakening and recovery, which was significantly longer than anticipated for an ultra-short-acting agent (Table 1).

**Table 1:** Summary of the outcome of the ONO-2745–04 trial

	< 24 h infusion	≥ 24 h infusion
Patients, n	42	7
Total administration time, h	12.90 ± 4.23	72.42 ± 28.68
Total dose, mg/kg	3.69 ± 2.25	39.50 ± 51.63
Time to awaking, min	71.71 ± 316.12	418.00 ± 395.95
Max. blood concentration, ng/mL	352.0 ± 194.7	2742.7 ± 2406.8

The data are mean ± standard deviation

Remimazolam is designed for rapid metabolic clearance by non-specific tissue esterases, predominantly CES-1, offering the benefit of non-accumulation. The ONO-2745-04 trial's results strongly suggested that in the context of severe critical illness and associated pathophysiology (e.g., organ dysfunction), the predicted rapid offset could not be reliably guaranteed. This potential for accumulation and delayed arousal, which posed an unacceptable risk to patient safety, led the Japanese developer to prioritize caution and suspend (later terminate) the program for the clinical development of remimazolam for long-term ICU sedation in Japan.

Although pilot studies such as the open-label dose-finding studies in China have demonstrated the feasibility of remimazolam and a rapid offset even after long-term use in postoperative mechanically ventilated patients [5, 7] (Table 2), the subsequent multicenter trial protocol [4] illustrates the rigorous requirements for clinical validation. To evaluate efficacy with a 10% non-inferiority margin regarding the percentage of time in the target range, the study required a large sample size of 728 patients. In this design, hard endpoints such as the duration of ventilation and 28-day mortality remain as secondary outcomes, reflecting the challenge of using them as primary measures for non-inferiority. In addition to these rigorous requirements, the complexity of trial designs can sometimes hinder the successful completion of clinical validation. A notable example is the Phase III study of remimazolam (CNS7056-011; EudraCT: 2014-004565-24). This randomized, single-blind, propofol-controlled study aimed to evaluate the efficacy and safety of remimazolam for general anesthesia in patients undergoing major cardiac surgery, with optional follow-up sedation in the ICU for up to 24 h. However, the trial was prematurely ended due to significant recruitment challenges. Despite intensive efforts, only 23 patients (18 in the remimazolam group and 5 in the propofol group) were enrolled globally between August 2015 and March 2016. This case underscores the critical need to balance scientific rigor with clinical feasibility when designing large-scale validation trials for novel sedatives in complex surgical environments.

**Table 2:** Summaries regarding remimazolam research in the ICU

Study (years)	Design	Sample size (RMZ/control)	Dosage	Duration	Primary outcomes	Main results
Tang et al. (2022) [7]	Phase I, open-label	20/-	0.05–0.2 mg/kg/h	Mean 10.6 h	Safety and feasibility	Successfully achieved RASS -1 to 0; No SAEs reported
Chen et al. (2022) [5]	Prospective, dose–response	23/-	Initial 0.2 mg/kg/h (titrated)	Up to 24 h	Effective dose (ED95)	ED95 was 0.25 mg/kg/h for target sedation. Stable hemodynamics
Tang et al. (2023) [6]	Randomized pilot (deep sedation)	30/30 (vs. Prop)	0.1–0.3 mg/kg/h	Median 42–45 h	Time in target RASS (-4 to -5)	Non-inferior to propofol in deep sedation; fewer hypotensive episodes
Yao et al. (2023) [3]	Prospective, observational	60/46 (vs. Prop/MDZ)	0.1–0.3 mg/kg/h	> 24 h (long-term)	ICU mortality	No significant difference in mortality; lower incidence of AKI in RMZ group
Tian et al. (2025) [12]	Randomized, prospective	40/40 (vs. Prop)	0.1–0.3 mg/kg/h	Procedural (intermittent)	Safety (organ function)	Lower impact on liver/kidney function and lipids compared to propofol
Grillot et al. (2025) [2]	Phase II, open-label	10/-	Up to 1.0 mg/kg/h	Median 10 h	Safety and PK	High clearance maintained; rapid awakening even in critically ill
ONO-2745–04 (Japan)	Phase II, randomized	14/16 (vs. Prop)	0.1–0.25 mg/kg/h	> 24 h	Safety and efficacy	Discontinued. Delayed awakening (≥ 1 h) observed in the > 24 h infusion subgroup
CNS7056-011	Phase III, randomized	18/5 (vs. Prop)	Up to 1.0 mg/kg/h	Up to 24 h	Sedation success	Discontinued. Low recruitment due to complex protocol

*Prop* propofol, *MDZ* midazolam

The Japanese experience underscores a common challenge in the development of drugs for use in ICU settings: a pharmacologically superior drug does not automatically translate into a clinically superior outcome under the scrutiny of a large, rigid Phase II/III trial. Variables such as subtle differences in sedation protocols, nurse-led titration practices, and the high baseline heterogeneity of the critically ill population may obscure the true pharmacokinetic advantages of remimazolam. This outcome is more than a reflection of the drug's pharmacological failure; it confirms the complexity of demonstrating statistically significant superiority over established, low-cost agents in a diverse ICU population.

## The pharmacokinetics of remimazolam in the critically ill: is accumulation a valid concern?

Remimazolam is distinct from its predecessor, midazolam, which relies heavily on hepatic cytochrome P450 isoenzymes [8]. The primary elimination pathway for remimazolam is rapid hydrolysis by CES-1 into an inactive metabolite, CNS 7054 [9]. This mechanism affords a very short elimination half-life (t_β1/2_) for remimazolam, typically < 10 min, plus a context-sensitive half-time that remains consistently short even after a prolonged infusion, demonstrating remimazolam's minimal tendency for parent drug accumulation [10].

However, the Japanese Phase II trial (ONO-2745-04) provided compelling, albeit concerning, evidence that the predictable short offset for remimazolam may not be reliably maintained in a subset of critically ill patients who receive a prolonged infusion. As mentioned above, the patients who received a continuous infusion for > 24 h experienced parent drug plasma concentrations that far exceeded predicted levels, resulting in significantly delayed awakening and recovery (requiring ≥ 1 h in all patients in that subgroup). This observation is critical because it suggests that, contrary to the basic pharmacological profile of remimazolam, the accumulation of the parent drug itself is a genuine risk factor for compromised recovery in the ICU setting.

This variability strongly suggests that severe critical illness and associated systemic inflammation or organ dysfunction can compromise the activity or quantity of CES-1, the key enzyme responsible for remimazolam's clearance. Although CES-1 activity is generally robust, factors such as systemic inflammatory response syndrome, a shock-induced reduction in tissue perfusion to key elimination sites, or severe nutritional compromise may reduce overall metabolic capacity, leading to a profound, though perhaps unpredictable, reduction in the parent drug's clearance. The delayed awakening observed in the patients in the ONO-2745-04 trial must therefore be attributable primarily to this impaired clearance of the active parent drug.

However, the question of accumulation is valid when considering the metabolite, CNS 7054. This carboxylic acid metabolite is pharmacologically inert, but its elimination occurs primarily through renal excretion. In the setting of severe critical illness, acute kidney injury (AKI) is common. When a patient's glomerular filtration rate is significantly compromised, the plasma concentration of the metabolite CNS 7054 can increase dramatically [9]. Although CNS 7054 is pharmacologically inactive and has low affinity for the gamma-aminobutyric acid (GABA)_A_ receptor, its substantial accumulation in patients with compromised glomerular filtration rates remains a characteristic of remimazolam’s PK profile in renal failure, albeit without anticipated clinical sedation effects.

Beyond clearance, hepatic and nutritional factors contribute to PK variability through distinct mechanisms. Unlike midazolam, which is highly dependent on cytochrome P450 isoenzymes, remimazolam's clearance via CES-1 demonstrates relative robustness in the presence of mild-to-moderate liver impairment [11]. However, in the setting of severe critical illness, two primary concerns emerge. First, extreme liver failure (e.g., acute liver failure or decompensated cirrhosis) could potentially compromise the synthesis and activity of CES-1, leading to an unpredictable reduction in clearance. Second, and perhaps more practically significant in the ICU, is severe hypoalbuminemia. Given remimazolam’s high plasma protein binding, decreased albumin levels are likely to significantly increase the unbound fraction of the drug. This change directly translates to an enhanced clinical effect and potential over-sedation, even if the total drug clearance rate remains stable.

The risk of compromised recovery with remimazolam in the critically ill is a multi-factorial concern. It involves the accumulation of the parent drug due to impaired CES-1 function secondary to systemic illness, the accumulation of the inactive metabolite CNS 7054 in renal impairment, and the enhanced drug effect caused by hypoalbuminemia. The ONO-2745-04 trial serves as a vital clinical warning: the advantage of a rapid offset is conditional on the patient’s physiological state. Close monitoring of renal function, protein levels, and depth of sedation is essential for safe titration in the ICU.

## Remimazolam in the ICU: international clinical experience and efficacy

Despite the challenges encountered in the Japanese development program, international research, particularly from China and Europe, has actively investigated the utility of remimazolam in various ICU settings. These efforts are driven by the drug’s highly favorable pharmacokinetic profile, which promises rapid and predictable recovery, a critical factor for early patient mobilization and a reduced duration of mechanical ventilation.

Early-phase and pilot studies, predominantly conducted in Chinese ICUs, established the initial feasibility of remimazolam for continuous intravenous infusion. Chen et al. [5] and Tang et al. [7] performed prospective dose–response studies in postoperative, mechanically ventilated patients. Their findings were instrumental in identifying the effective dose range required to achieve light-to-moderate sedation (RASS − 3 to 0). This groundwork confirmed that remimazolam can maintain consistent target sedation depths, which is crucial for minimizing over-sedation.

A key area of international focus has been the comparison of remimazolam with the current standard of care, propofol, for long-term sedation. Tang et al. conducted a randomized pilot study in patients requiring long-term mechanical ventilation [6]. Their study's primary outcome, the percentage of time spent by the patient within the target RASS range without requiring rescue sedation, showed no significant difference between the remimazolam and propofol groups. This suggests that remimazolam is non-inferior to propofol in terms of maintaining effective and titratable sedation control. Crucially, the rapid emergence of patients from sedation upon discontinuation of remimazolam was consistently noted, as a direct clinical manifestation of remimazolam's ultra-short context-sensitive half-time.

Further real-world evidence comes from observational studies. In a single-center, prospective, observational study comparing remimazolam against a combined propofol + midazolam group for long-term sedation (infusion lasting > 24 h), Yao et al. obtained critical reassurance concerning hard clinical outcomes [3]. Their analyses revealed no significant differences in ICU mortality, length of ICU stay, or 28-day mortality between the remimazolam and propofol + midazolam groups. These results, while requiring confirmation in larger-scale trials, support the initial premise that remimazolam can be safely integrated into the ICU armamentarium without negatively impacting major patient outcomes.

Beyond continuous maintenance, remimazolam’s unique properties make it highly suitable for procedural sedation in the ICU, where a rapid onset and prompt recovery are paramount. Tian et al. assessed remimazolam for procedural sedation in intubated patients undergoing blood tests and vital sign measurements [12]. Their comparison with propofol investigated various endpoints, including the impacts of remimazolam and those of propofol on organ function and the incidence of delirium, suggested a favorable profile of remimazolam for use during short, painful, or distressing procedures.

European efforts such as the Phase 2 open-label pilot study by Grillot et al. in critically ill patients [2] further validated the performance of remimazolam. That study's results confirmed the feasibility and safety of titrating remimazolam to target sedation depths (RASS − 2 to 0) for up to 24 h. Collectively, these international clinical investigations indicate that the formal-proof organ-independent pharmacokinetics of remimazolam translate into a highly manageable and effective sedative across various high-acuity scenarios in the ICU. A multicenter randomized non-inferiority trial protocol from China also continues to explore the use of remimazolam versus propofol during invasive mechanical ventilation, underscoring the ongoing international commitment to defining remimazolam's definitive roles [4].

## Remimazolam’s safety profile and adverse events in the ICU

The safety profile of remimazolam in critically ill populations is one of its most compelling attributes, offering distinct advantages over both propofol and midazolam, particularly concerning cardiovascular decompression and metabolic and long-term toxicity.

### Advantages of remimazolam in the ICU

#### Stable hemodynamics

One of the most compelling advantages of remimazolam over traditional ICU sedatives like propofol is its improved profile regarding hemodynamic stability. Several clinical trials have demonstrated that remimazolam induction is associated with a lower incidence and magnitude of a decrease in patients' mean arterial pressure (MAP) compared to propofol, especially in middle-aged and elderly patients and those undergoing high-risk procedures such as valve replacement surgery [13–15]. This superior profile is supported by direct mechanistic evidence: a combined study [16] reported that during induction, the decrease in the maximum rate of increase in left ventricular pressure (dP/dt max) was significantly smaller with remimazolam than with propofol (16.2% vs. 31.0%, p < 0.001). Furthermore, a preclinical ex vivo study in the same report showed that the concentration of remimazolam required to induce a 50% reduction in left ventricular developed pressure was approximately 16.7-fold higher than that of propofol, providing a fundamental physiological basis for remimazolam's superior hemodynamic stability.

The authors of a randomized controlled trial (RCT) [17] described remimazolam as a hemodynamically superior alternative to dexmedetomidine in older patients undergoing orthopedic surgery under spinal anesthesia. The study revealed that the remimazolam group had a significantly lower incidence of bradycardia compared to the dexmedetomidine group (9.5% vs. 61.9%, p < 0.001). Quantitatively, while the dexmedetomidine group experienced a marked decline in heart rate (HR), decreasing from a baseline of 68.6 ± 9.3 bpm to a nadir of 53.3 ± 7.6 bpm at 30 min post-administration, the remimazolam group maintained greater stability, with HR values remaining significantly higher throughout the procedure (e.g., 65.6 ± 8.7 bpm at 30 min; p < 0.05) [17]. Consequently, the remimazolam group required fewer interventions with atropine. This difference is attributed to dexmedetomidine's α2-adrenoceptor agonism, which centrally inhibits sympathetic outflow and inherently predisposes patients to dose-dependent bradycardia, whereas remimazolam’s profile avoids such pronounced vagal potentiation.

In contrast, remimazolam’s ultra-short-acting GABA_A_ receptor modulation and rapid esterase metabolism minimize cardiovascular depression and drug accumulation, leading to superior hemodynamic stability. This benefit was visually supported in the Wang et al. study by the serially assessed heart rate (HR), which was significantly higher in the remimazolam group from 15 min after the initiation of sedation onward [17]. Remimazolam’s faster offset facilitates a swifter return to baseline hemodynamics, which is a critical advantage over dexmedetomidine, as the use of dexmedetomidine often leads to persistently lower MAP and HR value extending into the patients’ time in the post-anesthesia care unit due to its longer half-life. These characteristics are vital in the ICU setting where patients are often hypovolemic, septic, and/or have pre-existing cardiovascular compromise.

The rapid and predictable clearance of remimazolam prevents the excessive or prolonged cardiovascular depression that can occur with the accumulation of dexmedetomidine, midazolam, or high-dose propofol. This ease of titration allows clinicians to maintain a stable depth of sedation for patients, without the profound drops in blood pressure that can lead to organ hypoperfusion [8].

#### Anti-inflammatory and organ-protective effects of remimazolam

A growing body of preclinical evidence suggests that remimazolam possesses intrinsic properties beyond mere sedation, including potential anti-inflammatory and organ-protective effects [18–22]. Although these observations were obtained primarily in animal models, the potential anti-inflammatory and organ-protective effects are particularly relevant for critically ill patients suffering from systemic inflammatory response syndrome and ischemia–reperfusion injury (IRI).

#### The mitigation of ischemia–reperfusion injury (IRI) by remimazolam

##### Cardioprotection

Remimazolam has been shown to attenuate myocardial IRI in animal models by inhibiting the NF-κB (nuclear factor kappa-light-chain-enhancer of activated B cells) signaling pathway in macrophages, thereby reducing inflammatory responses following ischemia [23].

##### Hepatoprotection

Several studies have indicated that remimazolam alleviates hepatic IRIs by multiple mechanisms, including the activation of the FOXO1/3 (Forkhead box O1 and O3 proteins) signaling pathway and the inhibition of the MAPK/ERK (mitogen-activated protein kinase–extracellular signal-regulated kinase) signaling pathway, which reduce oxidative stress, inflammation, and apoptosis in hepatocytes [21, 22, 24]. Recent in vivo and in vitro investigations further confirmed these mechanisms, demonstrating that remimazolam significantly prevents hepatic IRI damage by inhibiting the phosphorylation of p38 and ERK1/2, consequently reducing the release of inflammatory mediators such as tumor necrosis factor-alpha (TNF-α) and interleukin (IL)-6 [24].

##### Renoprotection

In a murine model of folic acid-induced AKI, remimazolam treatment alleviated kidney damage and reduced inflammation and fibrosis, suggesting a potential role for remimazolam in preventing the transition from AKI to chronic kidney disease by modulating macrophage activity [25].

##### Endotoxemia/sepsis

Remimazolam treatment was reported to improve survival in mice with lipopolysaccharide (LPS)-induced endotoxemia by protecting against the toxic effects of endotoxins, which highlights a potential benefit of remimazolam for patients with sepsis or septic shock [26].

#### Remimazolam’s effects on cognitive function and postoperative delirium (POD)

Minimizing the incidence and duration of delirium is a high-priority goal in critical care, as this complication is independently associated with patients' longer ICU stays, increased mortality, and poor long-term cognitive outcomes. Traditional, long-acting benzodiazepines such as midazolam are frequently implicated in the development and prolongation of postoperative delirium (POD) [27], which is due primarily to these drugs' active metabolites and erratic accumulation, especially in patients with organ dysfunction. Remimazolam represents a significant pharmacological advancement in this area; its unique ultra-short-acting profile, achieved via rapid metabolism by CES-1, provides exceptional control over the depth of sedation. Crucially, remimazolam's rapid clearance minimizes drug accumulation, thereby reducing the duration of drug exposure to the central nervous system, which is a key pharmacokinetic strategy for delirium prevention. This benefit, combined with the availability of the sedation-reversal agent flumazenil, makes remimazolam a highly titratable sedative with strong potential for reducing the incidence of POD.

Beyond its sedating properties, preclinical research strongly suggests that remimazolam possesses intrinsic anti-neuroinflammatory and neuroprotective effects. Multiple studies utilizing a rodent model of LPS-induced cognitive dysfunction have demonstrated that remimazolam effectively attenuates neuroinflammation, mitigates oxidative stress, and reverses corresponding behavioral and cognitive deficits [18, 19, 28]. Mechanistically, this protection appears to involve the suppression of M1 microglial activation and the upregulation of key anti-oxidative pathways, specifically the Nrf2/HO-1 signaling cascade [18, 19, 28]. This foundational evidence positions remimazolam as a potential pharmacological agent for mitigating the central nervous system component of systemic inflammatory states, such as sepsis-associated encephalopathy.

##### Reduced emergence delirium in children

A randomized controlled trial [29] demonstrated that remimazolam significantly reduced the incidence of emergence delirium (ED) in children undergoing laparoscopic surgery. Compared to the placebo group (44%), the incidence of ED (defined as a Pediatric Anesthesia Emergence Delirium (PAED) score ≥ 10) was significantly lower in both the remimazolam bolus group (15%; relative risk [RR], 0.33; 95% CI, 0.18–0.62; p < 0.001) and the continuous infusion group (10%; RR, 0.22; 95% CI, 0.11–0.46; p < 0.001). Furthermore, the severity of delirium was also reduced, with the peak PAED scores being significantly lower in the bolus (median 6) and infusion (median 5) groups compared to the placebo group (median 9, p < 0.001) [29]. The efficacy and safety of remimazolam for the induction and maintenance of general anesthesia in pediatric patients undergoing elective surgery have been rigorously confirmed, demonstrating its non-inferiority to propofol with respect to successful anesthesia and a comparable safety profile, marking remimazolam as a viable option for a broad pediatric population [30]. In addition, a dedicated randomized study confirmed that a single prophylactic dose of remimazolam administered at the end of surgery significantly reduced the incidence and severity of emergence delirium in children following tonsillectomy and adenoidectomy surgeries conducted with sevoflurane anesthesia [31].

##### Postoperative neurocognitive outcomes

Although definitive ICU data related to remimazolam are still emerging, some studies suggest a potentially favorable cognitive profile. Remimazolam's effects on cognition and neuropathology were investigated in a murine model of tibia fracture, and the results supported the exploration of the impact of remimazolam use on perioperative neurocognitive disorders [32]. In an examination of elderly patients, compared to the use of propofol, remimazolam showed potentially less disruption to the postoperative sleep rhythm and melatonin secretion, which are two factors associated with POD [33].

##### Early neurological assessment

The rapid offset of remimazolam facilitates a quicker recovery of consciousness after the cessation of an infusion, enabling an earlier neurological assessment and potentially shortening the durations of mechanical ventilation and ICU stays, providing a key strategy for delirium prevention [12].

### Disadvantages of and unresolved issues concerning remimazolam

#### Challenges with long-term sedation and optimal dosing

Despite its rapid elimination profile, remimazolam’s use for long-term sedation (> 24 h) in the ICU is still in the early stages of investigation, relying primarily on pilot studies and small cohorts.

#### Limited ICU experience

Although some pilot studies have explored the efficacy and safety of remimazolam for long-term mild-to-moderate sedation (RASS − 3 to 0) and deep sedation (RASS − 4 to − 5) in mechanically ventilated patients, large-scale, multicenter RCTs comparing remimazolam to standard ICU sedatives (propofol, midazolam, or dexmedetomidine) regarding critical outcomes (e.g., number of ventilator-free days, length of ICU stay, and mortality) are still needed [2, 3, 6, 34]. A non-inferiority RCT protocol for long-term sedation with remimazolam is currently underway, aiming to address this gap [4].

#### Optimal dosing strategy

The highly variable pharmacokinetics of critically ill patients means that even with a non-organ dependent drug, it remains difficult to determine the optimal, individualized continuous infusion rate [5, 7, 9]. A Phase I open-label, dose-finding study suggested a maintenance remimazolam dose starting at around 0.2 mg/kg/h for sedation in postoperative ICU patients [5], but generalized recommendations for a diverse ICU population are not yet established.

### Tolerance and addiction (abuse potential)

As a member of the benzodiazepine class, remimazolam carries the inherent risk of central nervous system-related side effects, particularly with prolonged use.

#### Tolerance

The development of tolerance (i.e., a diminished drug effect over time, requiring higher doses) is a known characteristic of benzodiazepines. Although remimazolam’s ultra-short action reduces the risk of drug accumulation, its long-term use in the ICU raises the concern of pharmacodynamic tolerance, which has been observed with other benzodiazepines during continuous infusion [35]. Specific data on the incidence and clinical impact of tolerance during prolonged remimazolam infusions in ICU patients are currently limited, necessitating caution and further study.

#### Addiction potential (habitual use)

Remimazolam acts via the GABA_A_ receptor, which is the same mechanism that confers the addictive potential (i.e., risk of habitual use and dependence) to all benzodiazepines. Although the uses of remimazolam in ICU settings is typically short-term and monitored (thus minimizing the risk of the development of long-term addiction), the risk of physical dependence and subsequent withdrawal symptoms upon abrupt cessation remain a significant concern if the drug is used for extended periods. Guidelines for weaning and managing withdrawal symptoms, similar to those for midazolam, should be considered for prolonged administrations of remimazolam in the ICU.

### Anti-tumor effects of remimazolam

#### Lack of clinical data

The potential for a direct anti-tumor effect of remimazolam is highly speculative and currently lacks any robust clinical evidence in the ICU setting. The issue of such an effect often arises in the context of sedation, as some literature suggests that certain anesthetics (e.g., propofol) might have neutral or even beneficial effects on cancer recurrence, whereas others (like some volatile agents) might be detrimental. Preclinical studies on remimazolam's oncological profile have obtained mixed and context-dependent results, highlighting the complexity of remimazolam's action. For example, in vitro studies of human colorectal cancer cells (HCT8) suggested that remimazolam may promote cellular proliferation and G1/S transition, raising potential concerns for certain cancer types [36]. Conversely, other in vitro research focusing on hepatocellular carcinoma (HCC) cells found that remimazolam induced cytotoxicity through multiple stress pathways and demonstrated a synergistic anti-cancer effect when combined with tyrosine kinase inhibitors (TKIs) [37].

Given these conflicting in vitro findings, it is impossible to draw any firm conclusions regarding remimazolam’s clinical effect on cancer progression or recurrence. Comprehensive, prospective clinical trials are therefore urgently needed to clarify the true oncological safety profile of remimazolam in surgical and critically ill cancer patients. At present, the potential anti-tumor properties of remimazolam should not be a factor in selecting a sedative for critically ill patients. More foundational research is required to ascertain any clinical relevance.

## The future role of remimazolam in ICUs in Japan

The paradigm of sedation in the ICU is rapidly evolving, moving away from deep, prolonged unconsciousness towards highly titratable, short-acting agents that facilitate early neurological assessment and mobility. In the demanding environment of Japanese critical care — where resource utilization and patient outcomes are under constant scrutiny — remimazolam, the ultra-short-acting benzodiazepine, represents a transformative pharmacological tool. Its unique pharmacokinetic and emerging pharmacodynamic profile positions it not merely as a replacement for older drugs such as midazolam but as a superior, next-generation option for managing critically ill patients.

The primary clinical advantage of remimazolam lies in its unparalleled hemodynamic stability. This is a crucial factor in the ICU, where patients frequently suffer from sepsis, hypovolemia, and/or pre-existing cardiovascular compromise. Compared to propofol, remimazolam exerts a significantly reduced negative effect on myocardial contractility, offering a fundamental physiological basis for its favorable hemodynamic profile [16]. Moreover, the outcomes of recent RCTs indicate that remimazolam is hemodynamically superior to dexmedetomidine, showing lower incidences of hypotension and bradycardia in vulnerable patient groups [17]. This stability, driven by rapid clearance via non-specific esterases, prevents the drug accumulation that leads to prolonged cardiovascular depression, allowing clinicians to maintain a stable sedation depth without the profound blood pressure drops that are associated with high-dose propofol, thus protecting vital organ perfusion [8].

Perhaps the most compelling argument for remimazolam’s adoption is its potential to mitigate delirium, which is a high-priority goal in Japanese critical care. Its ultra-short duration of action is a crucial pharmacokinetic strategy, minimizing the exposure of the central nervous system to the drug (a known risk factor for delirium), in sharp contrast to traditional benzodiazepines like midazolam. Moreover, compelling preclinical research supports an intrinsic organ-protective role for remimazolam, suggesting that remimazolam attenuates neuroinflammation, mitigates oxidative stress, and reverses cognitive deficits in systemic inflammatory states via pathways such as the Nrf2/HO-1 signaling cascade [28]. Clinically, this translates to tangible benefits, as evidenced by remimazolam’s proven ability to significantly reduce the incidence and severity of emergence delirium in pediatric populations, thus setting a strong precedent for the use of remimazolam in preventing ICU delirium among adult patients [31, 32].

However, as a newer agent, remimazolam's role requires continued investigation. The question of its long-term effects, particularly regarding tolerance and its oncological safety profile, remains complex. The preclinical in vitro findings are mixed, including a potential promotion of colorectal cancer cells' proliferation [36] but cytotoxicity in hepatocellular carcinoma cells [37]. Given the increasing complexity and number of cancer patients admitted to ICUs in Japan, comprehensive prospective clinical trials are vital to definitively establish the oncological neutrality or safety of remimazolam in this critical demographic.

In conclusion, remimazolam was developed as a promising sedative for ICU use, offering the potential for rapid recovery and hemodynamic stability due to its metabolism by CES-1. However, the discontinuation of its development for ICU sedation in Japan serves as a critical clinical warning. The ONO-2745-04 trial demonstrated that in the context of severe critical illness and prolonged infusion exceeding 24 h, remimazolam can lead to unpredictable accumulation and significantly delayed awakening.

The lessons learned from this failure are twofold. First, the pharmacological advantage of a rapid offset is not absolute; it is conditional upon the patient’s underlying physiological state and metabolic capacity. Clinicians must recognize that systemic inflammation, organ dysfunction, and severe hypoalbuminemia—common in the ICU—can compromise CES-1 activity and alter drug distribution, potentially nullifying the ultra-short-acting profile of the drug. Second, the discontinuation highlights the difficulty of implementing rigorous, complex protocols in the demanding ICU environment, as seen in the subsequent global trials.

While remimazolam remains a valuable tool for procedural sedation and short-term use, its application for long-term sedation in the critically ill requires a cautious, individualized approach. Close monitoring of the depth of sedation and a high level of vigilance regarding potential accumulation are essential when remimazolam is administered beyond the short term in the ICU.

## Data Availability

No datasets were generated or analyzed during the current study.

## References

[1] Hume NE, Zerfas I, Wong A, Klein-Fedyshin M, Smithburger PL, Buckley MS, et al. Clinical impact of the implementation strategies used to apply the 2013 pain, agitation/sedation, delirium or 2018 pain, agitation/sedation, delirium, immobility, sleep disruption guideline recommendations: a systematic review and meta-analysis. Crit Care Med. 2024;52:626–36. 38193764 10.1097/CCM.0000000000006178 PMC10939834

[2] Grillot N, Vourc’h M, Hourmant Y, Bouras M, Rozec B, Rouhani A, et al. A phase 2 open-label pilot study of remimazolam for sedation in critically ill patients. Anaesth Crit Care Pain Med. 2025;44:101510. 40174701 10.1016/j.accpm.2025.101510

[3] Yao Z, Liao Z, Li G, Wang L, Zhan L, Xia W. Remimazolam tosylate’s long-term sedative properties in ICU patients on mechanical ventilation: effectiveness and safety. Eur J Med Res. 2023;28:452. 37865799 10.1186/s40001-023-01440-9 PMC10590506

[4] Yang X, Tang Y, Du R, Yu Y, Xu J, Zhang J, et al. Long-term sedation with remimazolam besylate versus propofol in critically ill patients during invasive mechanical ventilation: a study protocol for a multicenter randomized non-inferior trial. Front Pharmacol. 2023;14:1139872. 37576823 10.3389/fphar.2023.1139872 PMC10416620

[5] Chen X, Zhang J, Yuan S, Huang H. Remimazolam besylate for the sedation of postoperative patients undergoing invasive mechanical ventilation in the ICU: a prospective dose‒response study. Sci Rep. 2022;12:19022. 36347892 10.1038/s41598-022-20946-6 PMC9643476

[6] Tang Y, Gao X, Xu J, Ren L, Qi H, Li R, et al. Remimazolam besylate versus propofol for deep sedation in critically ill patients: a randomized pilot study. Crit Care. 2023;27:474. 38049909 10.1186/s13054-023-04760-8 PMC10694930

[7] Tang Y, Yang X, Shu H, Yu Y, Xu J, Pan S, et al. Remimazolam besylate for sedation of postoperative patients in intensive care units: a phase I, open label, dose-finding study. Chin Med J (Engl). 2022;135:2134–6. 36191588 10.1097/CM9.0000000000002243 PMC9746757

[8] Schuttler J, Eisenried A, Lerch M, Fechner J, Jeleazcov C, Ihmsen H. Pharmacokinetics and pharmacodynamics of remimazolam (CNS 7056) after continuous infusion in healthy male volunteers: part I. Pharmacokinetics and clinical pharmacodynamics. Anesthesiology. 2020;132:636–51. 31972655 10.1097/ALN.0000000000003103

[9] Hu J, Zhao Y, Shao L, Zhang W, Wang H, Liu Y, et al. Pharmacokinetic properties and therapeutic effectiveness of remimazolam in ICU patients with mechanical ventilation: a preliminary study. Pharmacol Res Perspect. 2025;13:e70130. 40517312 10.1002/prp2.70130 PMC12167508

[10] Zhou J, Leonowens C, Ivaturi VD, Lohmer LL, Curd L, Ossig J, et al. Population pharmacokinetic/pharmacodynamic modeling for remimazolam in the induction and maintenance of general anesthesia in healthy subjects and in surgical subjects. J Clin Anesth. 2020;66:109899. 32585566 10.1016/j.jclinane.2020.109899

[11] Stohr T, Colin PJ, Ossig J, Pesic M, Borkett K, Winkle P, et al. Pharmacokinetic properties of remimazolam in subjects with hepatic or renal impairment. Br J Anaesth. 2021;127:415–23. 34246461 10.1016/j.bja.2021.05.027

[12] Tian Y, Li J, Jin M, Piao Y, Sheng J, Mei Z, et al. Procedural sedative effect of remimazolam in ICU patients on invasive mechanical ventilation: a randomised, prospective study. Ann Intensive Care. 2025;15:8. 39808218 10.1186/s13613-025-01431-5 PMC11732822

[13] Sekiguchi R, Kinoshita M, Kawanishi R, Kakuta N, Sakai Y, Tanaka K. Comparison of hemodynamics during induction of general anesthesia with remimazolam and target-controlled propofol in middle-aged and elderly patients: a single-center, randomized, controlled trial. BMC Anesthesiol. 2023;23:14. 36624371 10.1186/s12871-023-01974-9 PMC9830695

[14] Liu T, Lai T, Chen J, Lu Y, He F, Chen Y, et al. Effect of remimazolam induction on hemodynamics in patients undergoing valve replacement surgery: a randomized, double-blind, controlled trial. Pharmacol Res Perspect. 2021;9:e00851. 34390228 10.1002/prp2.851 PMC8363771

[15] Kuang Q, Zhong N, Ye C, Zhu X, Wei F. Propofol versus remimazolam on cognitive function, hemodynamics, and oxygenation during one-lung ventilation in older patients undergoing pulmonary lobectomy: a randomized controlled trial. J Cardiothorac Vasc Anesth. 2023;37:1996–2005. 37422336 10.1053/j.jvca.2023.06.027

[16] Yoshikawa Y, Oura S, Kanda M, Chaki T, Hirata N, Edanaga M, et al. Comparison of the negative effect of remimazolam and propofol on cardiac contractility: analysis of a randomised parallel-group trial and a preclinical ex vivo study. Clin Exp Pharmacol Physiol. 2024;51:e13840. 38302076 10.1111/1440-1681.13840

[17] Wang D, Liu Z, Zhang W, Li S, Chen Y, Jiang C, et al. Comparison of remimazolam versus dexmedetomidine on hemodynamics in older patients under lower extremity orthopedic surgery with spinal anesthesia: a randomized controlled trial. Drug Des Devel Ther. 2025;19:6037–46. 40687899 10.2147/DDDT.S504371 PMC12273724

[18] Zhou L, Shi H, Xiao M, Liu W, Wang L, Zhou S, et al. Remimazolam attenuates lipopolysaccharide-induced neuroinflammation and cognitive dysfunction. Behav Brain Res. 2025;476:115268. 39322063 10.1016/j.bbr.2024.115268

[19] Zhou Z, Yang Y, Wei Y, Xie Y. Remimazolam attenuates LPS-derived cognitive dysfunction via subdiaphragmatic vagus nerve target alpha7nAChR-mediated Nrf2/HO-1 signal pathway. Neurochem Res. 2024;49:1306–21. 38472553 10.1007/s11064-024-04115-x PMC10991060

[20] Liu Y, Liu C, Zhou Q, Lu H, Pan P. Comparison of remimazolam besylate and dexmedetomidine on stress response and immune balance in patients with acute respiratory distress syndrome. Ir J Med Sci. 2025;194:1797–803. 40794254 10.1007/s11845-025-04032-0

[21] Fang H, Zhang Y, Wang J, Li L, An S, Huang Q, et al. Remimazolam reduces sepsis-associated acute liver injury by activation of peripheral benzodiazepine receptors and p38 inhibition of macrophages. Int Immunopharmacol. 2021;101:108331. 34810122 10.1016/j.intimp.2021.108331

[22] Zhou B, Liu J, Jin L, Huang X. Remimazolam alleviates hepatic ischemia-reperfusion injury by activating FOXO1/3 signaling: remimazolam alleviates hepatic ischemia reperfusion injury. BMC Gastroenterol. 2025;25:283. 40263992 10.1186/s12876-025-03820-3 PMC12016092

[23] Xu H, Chen Y, Xie P, Lei T, Liu K, Liu X, et al. Remimazolam attenuates myocardial ischemia-reperfusion injury by inhibiting the NF-kB pathway of macrophage inflammation. Eur J Pharmacol. 2024;965:176276. 38113966 10.1016/j.ejphar.2023.176276

[24] Shi Y, Deng H, Zhang Z, Zhu X, Zeng Z. Remimazolam protects the liver from ischemia-reperfusion injury by inhibiting the MAPK/ERK pathway. BMC Anesthesiol. 2024;24:251. 39054453 10.1186/s12871-024-02641-3 PMC11270846

[25] Song J, Yu W, Chen S, Huang J, Zhou C, Liang H. Remimazolam attenuates inflammation and kidney fibrosis following folic acid injury. Eur J Pharmacol. 2024;966:176342. 38290569 10.1016/j.ejphar.2024.176342

[26] Liu X, Lin S, Zhong Y, Shen J, Zhang X, Luo S, et al. Remimazolam protects against LPS-induced endotoxicity improving survival of endotoxemia mice. Front Pharmacol. 2021;12:739603. 34867346 10.3389/fphar.2021.739603 PMC8641375

[27] Memtsoudis S, Cozowicz C, Zubizarreta N, Weinstein SM, Liu J, Kim DH, et al. Risk factors for postoperative delirium in patients undergoing lower extremity joint arthroplasty: A retrospective population-based cohort study. Reg Anesth Pain Med. 2019;1:100700. 10.1136/rapm-2019-100700 31302641

[28] Wei Y, Pan S, Zhou Z, Yang Y, Liu T, Chen J, et al. Remimazolam attenuated lipopolysaccharide-induced behavioral deficits and neuronal injury via activation of the Nrf2 pathway. Sci Rep. 2025;15:13784. 40258855 10.1038/s41598-025-95379-y PMC12012220

[29] Cai YH, Zhong JW, Ma HY, Szmuk P, Wang CY, Wang Z, et al. Effect of remimazolam on emergence delirium in children undergoing laparoscopic surgery: a double-blinded randomized trial. Anesthesiology. 2024;141:500–10. 38758221 10.1097/ALN.0000000000005077 PMC11323754

[30] Fang YB, Zhong JW, Szmuk P, Lyu YL, Xu Y, Qu S, et al. Safety and efficacy of remimazolam tosilate for general anaesthesia in paediatric patients undergoing elective surgery: a multicentre, randomised, single-blind, controlled trial. Anaesthesia. 2025;80:259–68. 39577009 10.1111/anae.16475

[31] Yang X, Lin C, Chen S, Huang Y, Cheng Q, Yao Y. Remimazolam for the prevention of emergence delirium in children following tonsillectomy and adenoidectomy under sevoflurane anesthesia: a randomized controlled study. Drug Des Devel Ther. 2022;16:3413–20. 36203819 10.2147/DDDT.S381611 PMC9531607

[32] Zhao J, Yu T, He R, Li M, Xia W, Lu Y. Effects of remimazolam and surgery on cognition in a tibia fracture mouse model. Int Immunopharmacol. 2024;143:113464. 39486180 10.1016/j.intimp.2024.113464

[33] Yaqiu L, Heng Z, Ruimin W, Xuri W. Effects of remimazolam and propofol on sleep rhythm and delirium after spinal surgery in elderly patients. Perioper Med. 2025;14:18. 10.1186/s13741-025-00500-4 PMC11817572 39934906

[34] Tang Y, Yang X, Yu Y, Shu H, Yuan Y, Liu H, et al. Remimazolam besylate versus propofol for long-term sedation during invasive mechanical ventilation: a pilot study. Crit Care. 2022;26:279. 36114552 10.1186/s13054-022-04168-w PMC9482181

[35] Elmati PR, Nagaradona T, Jagirdhar GSK, Surani S. Remimazolam in intensive care unit: potential applications and considerations. World J Crit Care Med. 2024;13:96877. 39253308 10.5492/wjccm.v13.i3.96877 PMC11372519

[36] Wang R, Li S, Hu H, Hou Q, Chu H, Hou Y, et al. Transcriptomic analysis and experiments revealed that remimazolam promotes proliferation and G1/S transition in HCT8 cells. Front Oncol. 2024;14:1345656. 38725628 10.3389/fonc.2024.1345656 PMC11079263

[37] Fan HL, Chen JL, Liu ST, Lee JT, Huang SM, Wu ZF, et al. Remimazolam induced cytotoxicity mediated through multiple stress pathways and acted synergistically with tyrosine kinase inhibitors in hepatocellular carcinoma. Redox Rep. 2025;30:2475696. 40053437 10.1080/13510002.2025.2475696 PMC11892054

